# Running Economy in the Vertical Kilometer

**DOI:** 10.3390/s23239349

**Published:** 2023-11-23

**Authors:** Pablo Jesus Bascuas, Héctor Gutiérrez, Eduardo Piedrafita, Juan Rabal-Pelay, César Berzosa, Ana Vanessa Bataller-Cervero

**Affiliations:** Facultad de Ciencias de la Salud, Universidad San Jorge, Autov. A-23 Zaragoza-Huesca, 50830 Villanueva de Gallego, Spain; pbascuas@usj.es (P.J.B.); hgutierrez@usj.es (H.G.); epiedrafita@usj.es (E.P.); jrabal@usj.es (J.R.-P.); avbataller@usj.es (A.V.B.-C.)

**Keywords:** performance monitoring, energy expenditure, human movement, trail running

## Abstract

New and promising variables are being developed to analyze performance and fatigue in trail running, such as mechanical power, metabolic power, metabolic cost of transport and mechanical efficiency. The aim of this study was to analyze the behavior of these variables during a real vertical kilometer field test. Fifteen trained trail runners, eleven men (from 22 to 38 years old) and four women (from 19 to 35 years old) performed a vertical kilometer with a length of 4.64 km and 835 m positive slope. During the entire race, the runners were equipped with portable gas analyzers (Cosmed K5) to assess their cardiorespiratory and metabolic responses breath by breath. Significant differences were found between top-level runners versus low-level runners in the mean values of the variables of mechanical power, metabolic power and velocity. A repeated-measures ANOVA showed significant differences between the sections, the incline and the interactions between all the analyzed variables, in addition to differences depending on the level of the runner. The variable of mechanical power can be statistically significantly predicted from metabolic power and vertical net metabolic COT. An algebraic expression was obtained to calculate the value of metabolic power. Integrating the variables of mechanical power, vertical velocity and metabolic power into phone apps and smartwatches is a new opportunity to improve performance monitoring in trail running.

## 1. Introduction

Over the past decade, there has been a significant increase in interest in sport field applications, driven by both users and technological companies. This interest has been propelled by advancements in the development of wearable sensors based on micro-electromechanical systems (MEMSs) [1]. These sensors find application during training sessions and sports competitions, serving the purpose of monitoring the internal training load [2], scheduling workouts and tracking the athlete’s fitness level progression. To achieve this objective, it is essential to develop automated assessment methods that analyze highly accurate variables capable of reflecting the physiological, metabolic, biomechanical and neuromuscular state of the athlete. Additionally, these methods should be easily implemented in low-cost sensors, such as inertial measurement units, linear transducers, potentiometers and global navigation satellite systems, among others [3].

Trail running races have increasingly gained the interest of amateur and professional runners around the world due to their great accessibility and low economic cost. Specifically, the vertical kilometer is a trend in trail running. In this modality, the athletes must complete a course of an approximately 1000 m vertical climb in a maximum of 5000 m total race length, although these parameters could change between different races, according to the rules of the International Skyrunning Federation [4].

Research on key performance parameters, both in road and trail running, has been a growing target of analysis by numerous health and sport science researchers. The aim of these studies is to understand in more depth those factors correlated with running performance to later be able to apply this knowledge in the creation of personalized trackers that can be implemented in phone apps and smartwatches. With technological advances, many scientists have developed new promising concepts whose assessment seems to be sensitive to physiological and biomechanical modifications during running and which may be suitable real feedback measures of performance and training monitoring in trail running and vertical kilometers. These concepts are the running economy, the net metabolic power, the mechanical vertical center of mass power, the net mechanical efficiency, the net metabolic cost of transport and the vertical net metabolic cost of transport.

Running economy is defined as the oxygen uptake (VO_2_) required to run a given distance or run at a given submaximal velocity [5]. This parameter can also be defined and calculated in energy terms as the amount of energy liberated per liter of oxygen, denominated in this case as net metabolic rate or power (Cmetab) (kcal·min^−1^·kg^−1^·or W·kg^−1^). It is calculated by measuring the steady-state consumption of oxygen (VO_2_) and the respiratory exchange ratio [6] and is considered a physiological determinant of endurance running. This variable is multifactorial, depending on metabolic, cardiorespiratory, biomechanical and neuromuscular factors [7], such as heart rate, minute ventilation, substrate utilization, muscle fiber type and core temperature, among many other variables, and is a new concept that reflects the physiological and neuromuscular state of the athlete [8]. It is currently considered more sensitive than VO_2_ itself when used to observe performance differences between runners [7,9].

The mechanical vertical power of the center of mass (Cmec) is defined as the external mechanical work performed to lift the body mass at each running stride, calculated by multiplying the vertical running velocity by the weight of the subject. Recent studies related to running power have found a linear relationship between running power and aerobic power (VO_2_ consumption) [10,11]. In addition, lower limb power is related to running spatiotemporal improvements (increased contact time), reduction in the energy cost of running [12] and reduction in the increase in energy cost of running due to fatigue in trail running [13]. Specifically, in vertical kilometers, runners must overcome extreme uphill running slopes, lifting the center of body mass in each step more than in level running by increasing the net mechanical work. This mechanism entails an increase in energy expenditure and a poorer mechanical advantage for producing force against the ground by the hip extensors [14].

Finally, from the previous concepts, the parameters of net mechanical efficiency, net metabolic cost of transport and vertical net metabolic cost of transport have emerged. The first authors to evaluate these parameters were Margaria et al., (1963) [15] and Minetti et al., (2002) [16]. They calculated the net metabolic cost of transport (both walking and running) (cost of walking (Cw) and cost of running (Cr)) by dividing the metabolic power or rate by running or walking velocity (vertical velocity for the vertical net metabolic cost of transport (VCw and VCr)). This parameter is a key factor in road running [4] and describes the amount of energy needed to transport a kilogram of body mass per unit of distance covered (kcal·kg^−1^·km^−1^ or J·kg^−1^·m^−1^). In their studies, Margaria et al., (1963) [15] and Minetti et al., (2002) [16] observed that the metabolic cost of running (Cr) was dependent on gradient and independent of speed, except for the steepest positive slopes (above 15% or 8.5°).

Based on these data, subsequent studies have found a great increase in Cr between slopes among runners, whose cause is still unknown, since uphill Cr correlates with neither level Cr nor with biomechanical parameters, such as stride frequency, stride length and body mass index [17]. Likewise, there is no correlation between either the initial Cr values or the changes in Cr values before and after the trail running race with performance time, in contrast to the observed correlation in road running [18]. The increase in Cr with a positive incline is due to an increase in power output and greater muscular activity at all joints, especially in the hip [19]. Unlike level running, where the center of mass behavior oscillates cyclically and both potential and kinetic energy fluctuation are in-phase during the stride [20], in uphill running above 15% (8.5°), positive work predominately lifts the center of mass and decreases the use of elastic energy (the stretch–shortening cycle mechanism disappears) and bouncing mechanisms [21,22]. Consequently, the metabolic demand increases, coinciding with an increase in blood lactate values and cardiorespiratory values [17,19,23].

In connection with the concepts of mechanical and metabolic power, Margaria et al., (1963) [15] and Minetti et al., (2002) [16] also introduced the concept of net mechanical efficiency (Eff) by explaining the ratio of these two variables. In their analysis, they observed that trained athletes were only 5–7% more efficient than non-athletes [15]. They predicted that mechanical efficiency was approximately 22–24% with positive slopes above 15% (8.5°) and 25% above 20% (11.3°), corresponding to concentric muscle contraction [15,16]. Peyré-Tartaruga et al., (2018) [24] proposed that overall efficiency in locomotion (walking and running) is determined by muscular efficiency, defined as the fraction of metabolic energy transformed into muscular mechanical work, and transmission efficiency, defined as the fraction of muscular mechanical work utilized as total work. However, for practical purposes, the concept net mechanical efficiency (Eff) is considered the fraction of metabolic power transformed into mechanical power or total work. These authors also contended that if the efficiency value was close to 25% (indicating pure concentric muscle efficiency), it would suggest good efficiency transmission. If the value exceeded 25%, it would indicate that passive elastic elements in series within muscles (fascial tissues) and tendons provided either the same or significant negative work.

Based on the studies analyzed to date, most research has been conducted on a treadmill in trail running, and any study of the vertical kilometer was executed through a field test. For these reasons, the present study aims to determine the correlation with performance in the previously mentioned concepts (Cmec, Cmetab, Cw, Cr, Vcw, VCr and Eff), as well as to observe the effect of fatigue on these concepts during the progress of a vertical kilometer field test.

## 2. Materials and Methods

### 2.1. Participants

Fifteen trained trail runners participated in the study (eleven males, four females). Demographic, anthropometric and training level data are presented in Table 1. All runners had been training regularly for more than 3 years, and none of them had a history of musculoskeletal injuries in the last year. Before the experiment, all subjects were informed about the objectives, benefits and risks of the investigation, and they signed an informed consent form. The experimental protocol received approval from the University Ethics Committee (Ref 005-19/20), and all procedures adhered to the principle of the Declaration of Helsinki.

### 2.2. Procedure

**Figure 1:**
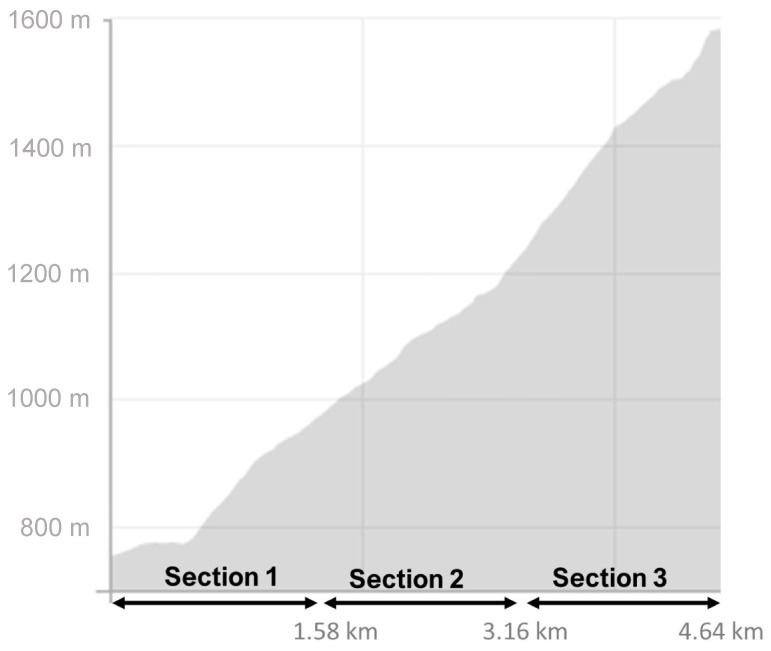
Vertical kilometer track. Race course divided into 3 sections of 1.58 km.

Each participant completed a vertical kilometer (VK) route spanning 4.64 km with a positive slope of 835 m. The vertical kilometer entails a continuous uphill test, comprising natural segments with varying positive inclinations ranging from 0° to 20° on this specific route. To facilitate analysis, the route was divided into three equal parts, each measuring 1.58 km, as illustrated in Figure 1. Within each of these segments, five sections with a constant slope were chosen (0°, 5°, 10°, 15°, and 20° positive slope). Each section had to last a minimum of 30 s to extract stable physiological data. Furthermore, to ensure data stability, only the central 20 s of each section were analyzed, excluding the initial and final portions of the positive slope.

### 2.3. Measurements

#### 2.3.1. Metabolic Data

Throughout the entire course, the runners were equipped with a portable gas analyzer (Cosmed K5 (Rome, Italy)) to assess cardiorespiratory and metabolic responses on a breath-by-breath basis. This measurement was facilitated by a turbine flowmeter attached to a properly fitted face mask. The gas analyzer was secured to the runner’s back using a harness, and the entire system weighted 900 g. To ensure time alignment, the analyzed parameters from the gas analyzer (including GPS data) were synchronized and stored in the data logger. Calibration of the Cosmed system was performed before each measurement, using a calibration syringe (3L) for the turbine. The oxygen (O_2_) and carbon dioxide (CO_2_) sensors of the gas analyzer were also calibrated to ambient air conditions (20.93% O_2_ and 0.03% CO_2_), along with delay calibration. Each experimental day commenced with determining the metabolic rate during a 10-min standing trial. Subsequently, rates of oxygen consumption (VO_2_) and carbon dioxide production (VCO_2_) were measured using the Cosmed K5 analyzer. For statistical analysis, the data for each slope and section were averaged over the selected 20-s intervals.

#### 2.3.2. Calculations

The calculation of mechanical vertical center of mass (COM) power (Watts/kg) utilized GPS velocity and incline, as expressed in (Equation (1)):

Mechanical vertical COM power = g × v × sin (θ) (1)

where θ represents the incline in degrees, and v is the instantaneous velocity in m/s.

Net metabolic power (Watts/kg) was calculated from running respiratory measurements using the Peronnet and Massicot equation [6], adjusted by subtracting the standing metabolic rate measured 10 min before the test. The calculation is outlined in (Equation (2)):

Net Metabolic power = ((16.89 × VO_2_ + 4.84 × VCO_2_)/kg) − standing metabolic rate (2)

The net mechanical efficiency was derived by dividing the mechanical vertical COM power by the net metabolic power, as illustrated in (Equation (3)) [25]:

Net mechanical efficiency = Mechanical vertical COM power/Net metabolic power(3)

The net metabolic cost of transport (J/kg/m) was computed by dividing the net metabolic power by the running velocity, representing the mean net metabolic cost per unit distance traveled parallel to the running surface. (Equation (4)) summarizes this calculation:

Net Metabolic COT = Net metabolic power/v (4)

The vertical net metabolic cost of transport (J/kg/m) was determined by dividing the net metabolic power by vertical velocity, factored by the mean net metabolic cost to ascend a vertical meter. (Equation (5)) outlines this computation:

Vertical Net Metabolic COT = Net metabolic power/v × sin (θ)(5)

### 2.4. Statistical Analysis

The following statistical analysis of the data was conducted:

- Normality testing: the Shapiro–Wilk test was used to assess the normality of the variables.
- Gender and performance level comparison: A T-student parametric test was employed to compare gender and performance level differences. The sample was divided into quartiles based on the final test time, and values from the first quartile were compared to the remaining quartiles.
- Comparison of assessed variables: A two-factor repeated-measures ANOVA was utilized to compare means across multiple analyzed variables. The analysis compared three sections and five positive slopes in each section. Before applying ANOVA, the Mauchly’s sphericity test was performed. If sphericity was rejected, the univariated F-statistic was used, adjusted with the Greenhouse–Geisser correction index. Bonferroni’s post hoc analysis was performed when significant differences were found for pairwise comparison.
- Statistical power and effect size determination: The statistical power (SP) and effect size (partial eta squared, ηp^2^) were determined. The effect size was categorized as trivial (ηp^2^ ≤ 0.01), small (0.01 ≤ ηp^2^ < 0.06), moderate (0.06 ≤ ηp^2^ < 0.14) or large (ηp^2^ ≥ 0.14) [26].
- Relationship analysis with final uphill time: Multiple regression and correlation models were calculated using an “intro” method. Mechanical vertical COM power was considered the dependent variable, and net metabolic power and vertical net metabolic cost of transport were the independent variables in the three VK sections. The entry and exit criteria were set at F probabilities greater than 0.05 and 0.10, respectively. The residual linearity and independence assumptions were checked with the Durbin–Watson test. The homoscedasticity was studied in a partial standardized residual-standardized prediction plot. The method of Bland and Altman was used to determine systematic bias and random error in the prediction model, as well as the lower and upper limits of agreement (1.96 × SD). The multicollinearity was estimated using a variance inflation factor (VIF), with values greater than 10 considered excessive. Influential cases (Cook’s distance > 1) and atypical cases (residual > 3 standard deviations) were removed from the analysis.
- A significance level of *p* < 0.05 was established. All statistical tests were conducted using the statistical package SPSS version 25.0 (SPSS, Chicago, IL, USA).

## 3. Results

Mean values for the three sections and five slope conditions are presented in Table 2. Regarding gender, no statistical differences were observed. Furthermore, when analyzing the aforementioned variables based on runner performance level (vertical kilometer final time) (Table 3), significant differences emerged between the first quartile and the remaining quartiles in the variables mechanical vertical COM power, net metabolic power, velocity and vertical velocity. On the other hand, no significant differences were identified in the variables net mechanical efficiency, net metabolic cost of transport and vertical net metabolic cost of transport.

A repeated-measures ANOVA revealed significant differences between sections, incline and the interaction of section x incline in all the variables presented in Table 4. These findings indicate a “Large” effect size of fatigue on all variables as the VK progresses. Additional distinctions are detailed in Table 5 through percentages.

**Figure 2:**
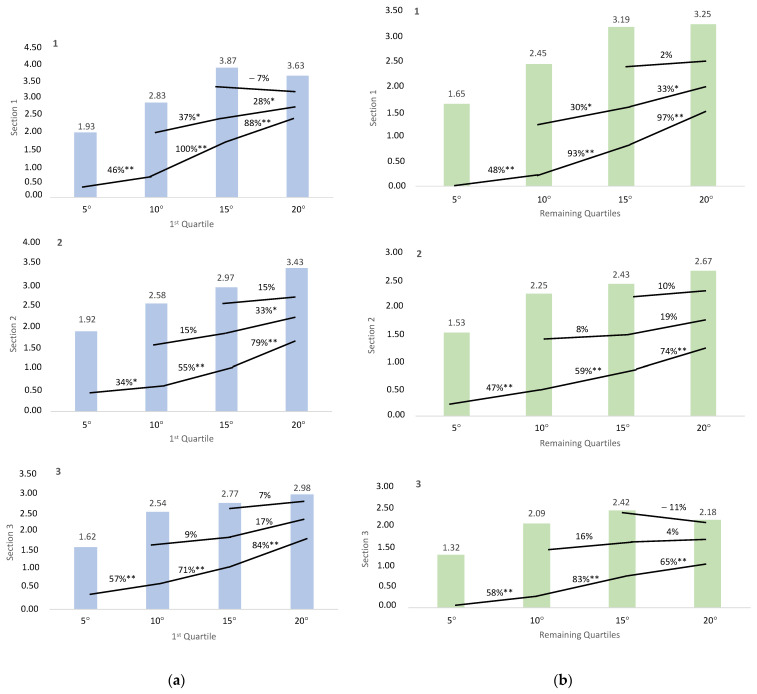
Percentage of change in mechanical vertical center of mass power between slopes. * *p*-value < 0.05. ** *p*-value < 0.001. (**a**) Percentage of change in the runners of the first quartile. The figures are arranged according to VK section (a1: first section; a2: second section; a3: third section). (**b**) Percentage of change in the runners of the remaining quartiles (b1: First section; b2: second section; b3: third section).

**Figure 3:**
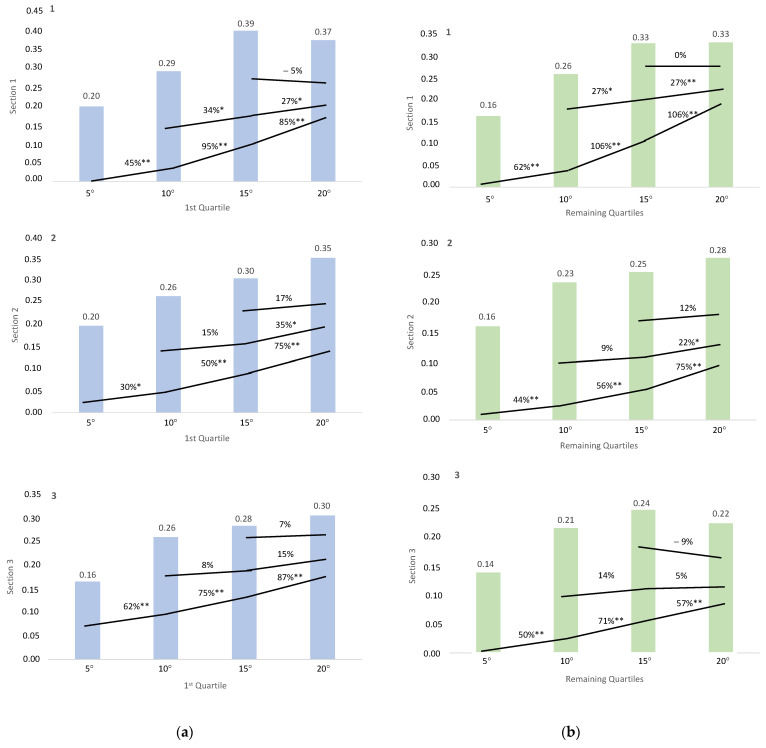
Percentage of change in vertical velocity between slopes. * *p*-value < 0.05. ** *p*-value < 0.001. (**a**) Percentage of change in the runners of the first quartile. The figures are arranged according to VK section (a1: first section; a2: second section; a3: third section). (**b**) Percentage of change in the runners of the remaining quartiles (b1: first section; b2: second section; b3: third section).

Conducting a two-way ANOVA with performance level as a factor (first quartile versus remaining quartiles), significant differences were only identified in mechanical vertical COM power (incline *p* < 0.001, SP = 0.967, ηp^2^ = 0.320) and vertical velocity (incline *p* < 0.001, SP = 0.972, ηp^2^ = 0.307). No significant differences were observed in net metabolic power, net mechanical efficiency, net metabolic COT, vertical net metabolic COT, and velocity. The percentage of change with corresponding *p*-values is depicted in Figure 2 and Figure 3.

A multiple regression analysis was conducted to predict mechanical vertical COM power from the remaining variables. The analysis revealed that the variable mechanical vertical COM power can be statistically significantly predicted using net metabolic power and vertical net metabolic cost of transport. This relationship held true across all sections and slopes.

The resulting model can be expressed algebraically as follows (Equation (6)):

(6) Mechanical Vertical COM power=αCmetab+βVCr+γ

where Cmetab represents net metabolic power (Equation (2)) and VCr stands for vertical net metabolic cost of transport (Equation (5)).

**Figure 4:**
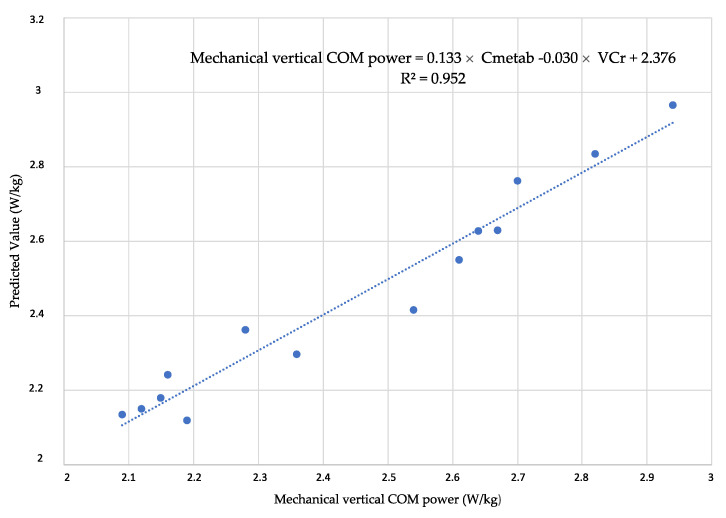
Scatter plot of the multiple linear regression model. Each data point represents the value of a subject in the study.

**Figure 5:**
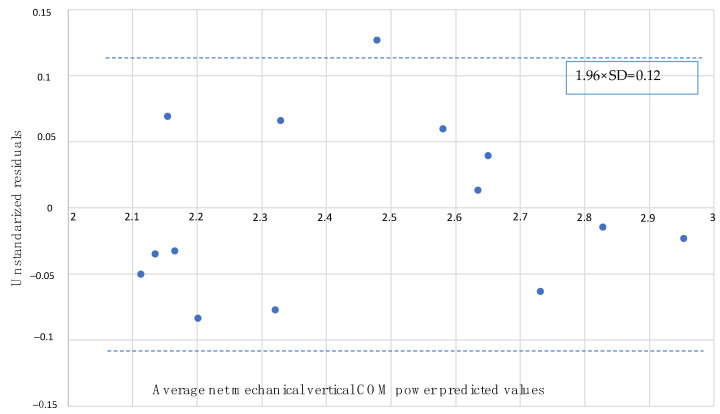
Bland–Altman plot of the multiple linear regression model. Each data point represents the value of a subject in the study.

The adjusted R^2^ of the multiple linear regression indicates that 94% of the variation in mechanical vertical COM power is explained by the proposed model (R^2^_adjusted_ = 0.942). The scatter plot for this model is illustrated in Figure 4. The model reached a significance level of *p* < 0.001. All variables included in the model exhibited a significance level below 0.001, suggesting their retention in the considered model. The Durbin–Watson test fell within the critical interval (1 < D–W < 3), allowing the acceptance of residual linearity and independence assumptions. However, Bland and Altman plots (Figure 5) revealed randomly distributed residuals concerning the average net mechanical vertical COM power predicted values. Only one value outside ±1.96 × SD was observed, and the residuals exhibited normal distribution based on the Shapiro–Wilk test (SW = 0.941; *p* = 0.434). All values presented a variance inflation factor (VIF) lower than 10 units. Therefore, the multicollinearity assumption is satisfied.

The results of the Bland–Altman analysis indicate the absence of systematic biases and random errors in our regression model, attributed to the randomness of the scatterplot dispersion and the absence of outliers.

Based on these results, the following prediction equations are derived (Equations (7) and (8)):

(7) Mechanical Vertical COM power=0.133×Cmetab−0.030×VCr+2.376

(8) $$Net\;metabolic\;power=\frac{V\times g\times sin\theta -2.376}{0.133-0.030\times {\left(V\times g\times sin\theta \right)}^{-1}}$$

Through mathematical calculation, the obtained algebraic expression allows us to calculate the value of net metabolic power solely from the subject’s vertical velocity (parallel velocity × sin θ (positive slope)).

## 4. Discussion

Metabolic efforts in trail running have recently become a significant focus of research, with studies conducted in both ultra-distance events and short trail running. In the majority of these studies, simulations of race slopes have been conducted using treadmill tests [27,28,29]. However, the metabolic demand appears to differ when the test is conducted outdoors, potentially making it a more suitable method [30]. To our knowledge, there are no metabolic studies during a vertical kilometer field test simulating a real race.

For these reasons, the primary objective of this study was to evaluate new concepts such as mechanical vertical COM power, net metabolic power, net metabolic cost of transport, vertical net metabolic cost of transport, and net mechanical efficiency during a real outdoor vertical kilometer field test, examining their changes with fatigue and the performance level of the athletes. The secondary goal was to analyze their relationships with the final time of the test.

### 4.1. Vertical Kilometer Performance Analysis

The T-test results showed no significant differences between genders, while revealing distinctions based on the subjects’ performance level, as despicted in Table 3.

Concerning the mean value differences between the first quartile and the remaining quartiles, better mean values were observed in top-level runners across all sections and inclines, achieving higher values in mechanical vertical COM power, net metabolic power, velocity and vertical velocity. The results suggest that better runners can apply more force and achieve greater vertical velocities as the slope increases. These disparities in power and vertical velocity persist throughout the entire duration of the VK. These outcomes align with expectations, as several researchers have observed that uphill running requires an increase in net mechanical work to increase the potential energy of the body, with concurrent increases in parallel propulsive force peaks and impulses with positive grades [31], since the bouncing mechanism gradually disappears as speed and slope increase [22]. The hip and knee joints are identified as the primary contributors to the augmented mechanical power [14,32]. Additionally, in short trail running, it has been observed that local endurance of knee extensors, assessed through repeated maximal concentric contractions, is a key performance factor in uphill running sections [33].

Net metabolic power reflects the instantaneous energy requirement for running, and it has been observed to increase linearly with speed in VK runners [34], attributable to the rise in O_2_ consumption and CO_2_ production. The higher metabolic power values among first quartile athletes are primarily explained by their greater velocity, stemming from either enhanced cardiorespiratory development or greater strength and power values. Moreover, the same study suggest that running is more efficient than walking above 0.8 m/s [34]. This reference value is crucial, as first-quartile runners could maintain speeds greater than 0.8 m/s with 20° positive grade in the section 3 of our VK test, while the remaining quartiles’ runners could not. This decision to walk instead of running may partly account for the observed difference in test performance.

Regarding the remaining variables, no significant differences were found based on performance level. Our results align with other studies where no differences were identified in the cost of running [35], and only a 5–7% difference in efficiency values [15] was observed among trail runners of different levels. The minimal variation in the net metabolic cost of transport in a real VK race could indicate that, despite first-quartile runners exhibiting higher metabolic power, their ability to attain higher speeds resulted in comparable cost of transport. This observation implies that net mechanical vertical COM power and net metabolic power may serve as more informative indicators of trail running performance compared to net metabolic cost of transport, as suggested by the existing literature [35]. These variables could prove more suitable for real-time tracking outdoors, utilizing potentiometers [36] or mobile applications, or for analyzing average values in both men and women to observe changes with training.

### 4.2. The Impact of Fatigue on the Vertical Kilometer

Analyzing the impact of fatigue throughout the progression of the VK (Table 4), we observed a deterioration in mean values across all monitored variables, occurring with all slopes, particularly notable between the first section and subsequent sections, and to a lesser extent between the second and third sections. The changes were more pronounced with steeper inclines (20°). There was a reduction in velocity and vertical velocity, possibly associated with the diminished ability to apply force (indicated by lower mechanical vertical COM power values). This reduction was more significant between the first and third sections, especially with 15° and 20° inclines, which are the most demanding due to lower use of elastic energy [21,37] and biomechanical changes during the transition from running to walking [38].

This power loss could stem from central fatigue (decreased amplitude and frequency of motor unit recruitment) or peripheral fatigue (alterations in potential transmission along the sarcolemma, excitation–contraction coupling and actin–myosin myofilament interaction) [39]. Both types of fatigue might be implicated based on previous findings in ultra-trail running [39,40,41,42,43].

Decreases in mechanical vertical COM power values could be attributed to fatigue in both plantar flexors and knee extensor muscles. Recent studies suggest that central fatigue tends to affect knee extensors more, while peripheral fatigue affects the plantar flexors [39,41,44]. However, caution is warranted in applying these conclusions to the VK, as these data were observed after an ultra-marathon.

A potential factor contributing to the onset of fatigue, particularly of central origin as posited by the central command theory [45], is muscle damage and inflammation. However, Pokora et al., (2014) [46] did not observe changes in creatine kinase (a marker of muscle damage) after 1 h of uphill running (10°) at 60%VO_2_max. Therefore, investigating muscle damage as a cause of fatigue in uphill running requires further exploration [46].

Decreases in metabolic power values were also observed, possibly caused by impairments in running biomechanics (such as increased step frequency, ankle joint changes and duty-free alterations) [39], arising from neuromuscular fatigue and behavioral changes in runners, especially with 15° and 20°inclines, choosing gaits that minimize metabolic cost [47].

Concerning net metabolic COT and vertical net metabolic COT, both continuously increased across all sections with steeper uphill inclines due to greater loss of velocity than metabolic power values as the test progressed. This suggests that neuromuscular, rather than cardiorespiratory factors, may be the primary contributors to the decline in performance in the VK. These increases align with observations in the literature after short-distance running races [48,49], 1 h of treadmill running [50] and the vertical kilometer [34]. Multiple reasons have been proposed for this increase in COT. Firstly, the steep inclines of the VK, coupled with a decrease in velocity, induce changes in running biomechanics, such as decreased step length, increased non-optimal step frequency, mid- to fore-foot strike patterns, and decreased leg stiffness, all associated with increased COT [9,51,52,53]. Prolonged running step contact times (“Groucho running” pattern concept) [54] could impair spring-like bouncing and elevate the COT due to changes in potential-kinetic energy savings [34,55]. These biomechanical changes may be induced by neuromuscular fatigue (reflected in decreased mechanical power) [39,56] or serve as a protective mechanism to reduce running impacts [57].

Regarding net mechanical efficiency changes, this variable decreased due to greater losses in mechanical power than metabolic power. The substantial and continuous losses in mechanical power could signify a decrease in workload due the loss of velocity, providing a significant limitation to performance due to the inability to utilize maximum metabolic potential. This theory is supported by data from Ettema et al., (2009) [58], who stated that power output is the main determinant of efficiency (more power leads to more efficiency and vice versa), owing to a greater utilization of metabolic power in running. The imbalance between mechanical power and metabolic power, resulting in a decrease in net mechanical efficiency, could be attributed to decreased energy transduction (due to decreased speed and stretch-shortening cycle) coupled with an increase in respiratory cost [24].

### 4.3. Examining Fatigue Effects Based on Runners’ Performance Levels

When examining the impact of fatigue based on the runners’ performance levels, we observed differential changes in only two variables, namely mechanical vertical COM power (Figure 2) and vertical velocity (Figure 3). Notably, elite runners demonstrated a better ability to sustain power values across all slopes, particularly evident with 10° and 20° inclines, resulting in more pronounced differences in power values between slopes. This phenomenon suggests their enhanced capability to exert force consistently across all slopes throughout the entire race. Similarly, top-level runners exhibited a superior ability to maintain vertical velocity values across all inclines, likely attributable to their heightened application of force throughout the entire VK. These findings align with prior research indicating a significant correlation between performance in short trail running races and neuromuscular capacity, as assessed by isometric knee extensor muscle torque, maximal theoretical force and maximal power from the force–velocity curve [59]. This underscores the importance of incorporating resistance training [60], uphill interval running training [61] and pulled running training [62] to enhance power and neuromuscular function [39,62] in runners. Additionally, it emphasizes the significance of monitoring these two variables using apps that measure speed and incline or smartwatches, which are increasingly employed in outdoor races and training sessions.

### 4.4. Metabolic Power Calculation

The outcomes of the multiple regression analysis revealed that 94% of the variance in mechanical vertical COM power during the VK test could be accounted for by net metabolic power and the vertical metabolic COT. This substantial explanation is primarily attributed to the fact that these two variables elucidate the vertical velocity, a key component of mechanical vertical COM power. From the derived equation (Equation (6)), three coefficients sensitive to the progression of the test and inclination were obtained (Table 6). These coefficients are likely subject to variations depending on the characteristics of the uphill test, such as slope, section lengths and their interaction. This observation is consistent with our study’s results, where net metabolic power levels exhibited changes due to slope and fatigue. The findings of this regression analysis suggest that, once an ascent has been characterized, net metabolic power can be estimated based on the runner’s vertical velocity. Consequently, a reliable equation (Equation (8)) was established from the multiple regression to calculate the runner’s metabolic power during a VK field test. This equation utilizes only the vertical velocity and the coefficients found in the model, eliminating the need for expensive portable gas analyzers. The ease of analysis with common devices like phones, smartwatches and GPS is a notable advantage [63]. These results align with the increasing interest among researchers to determine metabolic power during actual competitions in various sports. This pursuit aims to enhance the understanding of the real workload for athletes, thereby improving training methods and periodization [64,65,66,67].

Our formula, combined with the VO_2_ submax at 30° formula developed by Giovanelli et al. [38], can serve as a valuable tool for characterizing VK runners based on easily measured variables in a real field test.

The study results offer novel insights into the significance of utilizing mechanical power, metabolic power and vertical velocity variables for performance analysis in vertical kilometer runners, regardless of gender. Furthermore, it underscores their susceptibility to impairment due to the influence of fatigue. These findings align with the increasing interest in acquiring high-quality information on athletes’ internal load through the progressive improvement of technology and data analysis methods [3]. Moreover, it opens up the possibility of conducting further research to deeper analyze these variables across various running modalities and both cyclic and acyclic sports.

A major strength of the present study lies in the simplicity with which these variables can be implemented in any existing wearable sensor on the market that utilizes IMUs and GNSS to calculate real-time velocity, accelerations, anthropometric data and terrain characteristics. These data facilitate the calculation of key parameters, eliminating the need for athletes and coaches to undergo time-consuming and fatiguing tests and allowing data collection during training and competition [2].

Finally, possessing a comprehensive understanding of key variables within each sporting context is crucial. This clarity is essential for precise data collection, enabling researchers and companies to save a significant amount of time developing software and sensors [2].

As future lines of research, it would be interesting to validate the metabolic power formula and continue studying runners through real field tests.

## 5. Limitations

The main limitation of the study is the small sample size, with only 11 male and 4 female participants. This limitation arose from the technical complexity and time cost associated with conducting the analyses in a true vertical kilometer field test. Future extensive analyses with a larger and more diverse sample should be conducted, particularly for the reliability and validity assessment of the metabolic power formula identified in this study.

Additionally, the absence of anthropometric analysis to determine the body fat percentage and the level of lower limb muscle mass among the runners represents another limitation. This lack of information prevents readers from gaining insights into the subjects’ fitness levels, which would provide better context for the study’s findings. Notably, individuals with lower body fat percentages and higher levels of leg muscle mass are often observed to perform better in trail running tests. Consequently, future studies should incorporate analyses of these parameters. Lastly, another limitation is the absence of a pre-vertical kilometer maximum treadmill test to assess the physiological condition of the runners, as well as a strength test to gauge their neuromuscular level. These factors are also crucial for race performance and should undergo thorough examination in future studies.

## 6. Conclusions

The study results revealed significant differences in the mean values of variables such as velocity, vertical velocity, mechanical vertical COM power and net metabolic power when comparing top-level runners to low-level runners during a vertical kilometer field test. Additionally, all analyzed variables were affected by fatigue as the test progressed, showing significant differences in how fatigue altered mechanical vertical COM power and vertical velocity when comparing top-level to low-level runners. A multiple regression analysis demonstrated that 94% of the mechanical vertical COM power during the vertical kilometer test could be explained by net metabolic power and vertical net metabolic cost of transport. Subsequently, a reliable equation was derived from the multiple regression to calculate each runner’s metabolic power during a vertical kilometer field test, utilizing only the vertical velocity and the coefficients identified in the model. These findings present an opportunity to explore new variables correlated with performance in short trail running, particularly in vertical kilometer races. These new variables are sensitive to performance disparities, exhibit changes with fatigue and are applicable to both male and female athletes. Importantly, they can be easily measured through apps, smartwatches, foot-pod potentiometers and GPS.

## Figures and Tables

**Table 1:** Demographic, anthropometric and training level data.

	Men	Women
**Age (years)**	22–38 *	19–35 *
	28.4 ± 5.11	27.7 ± 6.70
**Height (cm)**	174 ± 4.54	163 ± 2.36
**Body mass (kg)**	69.8 ± 5.56	54 ± 4.08
**BMI (kg/m^2^)**	22.8 ± 1.63	20.2 ± 1.01
**Running training duration per session (min)**	52 ± 7.58	60 ± 21.6
**Running training frequency per week (days/week)**	4.40 ± 1.14	4.75 ± 1.26
**Pre-test heart rate (bpm)**	73.8 ± 10.7	79.5 ± 3.31
**HR change (%)**	16.1 ± 4.99	61.2 ± 56.6
**VO_2_ peak (mL/kg/min)**	65.8 ± 7.00	57.9 ± 6.61

Values: Mean ± SD. BMI: body mass index. HR change: percentage change in heart rate during the vertical kilometer test. VO_2_ peak achieved in the vertical kilometer test. *: age range of participants.

**Table 2:** Descriptive data of values in the three sections and five slope conditions.

	Section 1					Section 2					Section 3				
	0°	5°	10°	15°	20°	0°	5°	10°	15°	20°	0°	5°	10°	15°	20°
**Velocity (m/s)**	3.42 ± 0.39	1.98 ± 0.34	1.52 ± 0.19	1.35 ± 0.21	1.00 ± 0.13	2.26 ± 0.38	1.95 ± 0.30	1.40 ± 0.24	1.03 ± 0.14	0.88 ± 0.14	2.39 ± 0.60	1.67 ± 0.27	1.33 ± 0.19	1.00 ± 0.22	0.73 ± 0.16
**Vertical velocity (m/s)**	0 ± 0	0.17 ± 0.03	0.26 ± 0.03	0.35 ± 0.05	0.34 ± 0.05	0 ± 0	0.17 ± 0.03	0.24 ± 0.04	0.27 ± 0.04	0.30 ± 0.05	0 ± 0	0.14 ± 0.02	0.23 ± 0.03	0.26 ± 0.06	0.25 ± 0.06
**RER**	0.94 ± 0.08	0.82 ± 0.08	0.88 ± 0.10	0.90 ± 0.10	0.81 ± 0.08	0.82 ± 0.08	0.81 ± 0.09	0.81 ± 0.08	0.80 ± 0.08	0.80 ± 0.08	0.79 ± 0.08	0.80 ± 0.08	0.78 ± 0.08	0.79 ± 0.07	0.80 ± 0.08
**Mechanical vertical COM power (W/kg)**	0 ± 0	1.69 ± 0.29	2.58 ± 0.32	3.42 ± 0.54	3.38 ± 0.47	0 ± 0	1.67 ± 0.26	2.37 ± 0.40	2.62 ± 0.36	2.94 ± 0.48	0 ± 0	1.42 ± 0.23	2.25 ± 0.33	2.54 ± 0.55	2.46 ± 0.55
**Net metabolic power (W/kg)**	17 ± 2.41	17.4 ± 3.10	18.8 ± 2.97	18.7 ± 2.80	17.5 ± 2.69	16.3 ± 2.89	16.4 ± 2.84	16.3 ± 2.93	16.5 ± 2.82	16.3 ± 2.97	15.1 ± 3.33	16 ± 2.92	16.3 ± 2.65	16.7 ± 2.77	16.8 ± 2.91
**Net mechanical efficiency**	0 ± 0	9.88 ± 1.54	13.9 ± 2.15	18.6 ± 3.10	19.5 ± 2.83	0 ± 0	10.3 ± 1.66	14.8 ± 2.91	16 ± 1.92	18.1 ± 2.05	0 ± 0	9.03 ± 1.37	14.0 ± 1.75	15.6 ± 4.48	14.7 ± 2.23
**Net metabolic cost of transport (J/kg/m)**	5.01 ± 0.72	8.84 ± 1.37	12.4 ± 1.81	14.0 ± 2.36	17.4 ± 2.16	7.26 ± 0.97	8.47 ± 1.19	11.8 ± 2.15	16.0 ± 1.96	18.7 ± 2.03	6.77 ± 2.47	9.66 ± 1.39	12.3 ± 1.54	17.2 ± 4.19	23.4 ± 3.72
**Vertical net metabolic cost of transport (J/kg/m)**	0 ± 0	101.6 ± 15.7	71.9 ± 10.5	54.2 ± 9.15	51.0 ± 6.32	0 ± 0	97.3 ± 13.6	68.5 ± 12.4	62.1 ± 7.59	54.8 ± 5.93	0 ± 0	111.1 ± 15.9	71.3 ± 8.92	66.8 ± 16.2	68.5 ± 10.9

Values: mean ± standard deviation. RER: respiratory exchange rate; COM: center of mass.

**Table 3:** Differences in values in the three sections and five slope conditions between first quartile and remaining quartiles.

		Section 1					Section 2					Section 3				
		0°	5°	10°	15°	20°	0°	5°	10°	15°	20°	0°	5°	10°	15°	20°
**Vertical velocity (m/s)**	1st quartile (*n* = 5)	0 ± 0	0.20 ± 0.02 *	0.29 ± 0.02 *	0.39 ± 0.06 *	0.37 ± 0.02 *	0 ± 0	0.19 ± 0.01 *	0.26 ± 0.04	0.30 ± 0.02 *	0.35 ± 0.03 **	0 ± 0	0.16 ± 0.02 *	0.26 ± 0.03 *	0.28 ± 0.02 *	0.30 ± 0.01 **
	Remaining quartiles (*n* = 9)	0 ± 0	0.16 ± 0.02	0.26 ± 0.03	0.33 ± 0.04	3.33 ± 0.05	0 ± 0	0.16 ± 0.02	0.23 ± 0.04	0.25 ± 0.03	0.28 ± 0.03	0 ± 0	0.14 ± 0.02	0.21 ± 0.02	0.24 ± 0.06	0.22 ± 0.04
**Velocity (m/s)**	1st quartile	3.74 ± 0.23 *	2.26 ± 0.27 *	1.67 ± 0.15 *	1.53 ± 0.22 *	1.10 ± 0.07 *	2.64 ± 0.14 *	2.25 ± 0.15 *	1.52 ± 0.24	1.17 ± 0.09 **	1.02 ± 0.09 *	2.79 ± 0.24 *	1.90 ± 0.24 *	1.50 ± 0.20 *	1.10 ± 0.68 *	0.89 ± 0.03 **
	Remaining quartiles	3.33 ± 0.41	1.86 ± 0.28	1.49 ± 0.22	1.27 ± 0.15	0.96 ± 0.14	2.10 ± 0.32	1.83 ± 0.27	1.35 ± 0.22	0.98 ± 0.12	0.81 ± 0.09	2.22 ± 0.61	1.58 ± 0.23	1.23 ± 1.10	0.94 ± 0.25	0.65 ± 0.13
**Mechanical vertical COM power (W/kg)**	1st quartile	0 ± 0	1.92 ± 0.23 *	2.82 ± 0.25 *	3.87 ± 0.56 *	3.62 ± 0.23 *	0 ± 0	1.92 ± 0.13 *	2.58 ± 0.41 *	2.97 ± 0.24 *	3.43 ± 0.32 **	0 ± 0	1.62 ± 0.20 *	2.54 ± 0.35 *	2.77 ± 0.17	2.98 ± 0.12 **
	Remaining quartiles	0 ± 0	1.56 ± 0.24	2.44 ± 0.28	3.19 ± 0.37	3.25 ± 0.53	0 ± 0	1.53 ± 0.21	2.25 ± 0.37	2.43 ± 0.26	2.66 ± 0.30	0 ± 0	1.32 ± 0.18	2.09 ± 0.19	2.42 ± 0.65	2.17 ± 0.48
**Net metabolic power (W/kg)**	1st quartile	19 ± 0.64 *#	20.1 ± 2.62 *#	22 ± 1.10 **#	21.6 ± 1.66 **#	20.1 ± 1.32 *#	19.5 ± 0.93 **#	19.6 ± 1.61 **#	19.7 ± 1.35 **#	19.7 ± 1.24 **#	19.8 ± 1.46 **#	18.6 ± 2.16 **#	19.4 ± 1.42 **#	19.3 ± 1.24 **#	19.9 ± 1.11 **#	20.2 ± 1.46 **#
	Remaining quartiles	15.9 ± 2.31	15.8 ± 2.21	17.1 ± 2.06	17.1 ± 1.81	16 ± 2.08	14.5 ± 1.75	14.6 ± 1.31	14.4 ± 1.28	14.8 ± 1.52	14.4 ± 1.19	13.1 ± 1.88	14.1 ± 1.13	14.6 ± 1.25	14.9 ± 1.42	14.9 ± 1.25

Values: mean ± standard deviation. COM: center of mass. * *p*-value < 0.05. ** *p*-value < 0.001. #: strong effect size (g Hedges > 0.8).

**Table 4:** Repeated-measures ANOVA results.

		*p*-Value	Power (SP)	Effect Size (ηp^2^)	
**Vertical velocity (m/s)**	Section	<0.001	1	0.779	Large
	Slope	<0.001	1	0.973	Large
	Interaction	<0.001	1	0.463	Large
**Velocity (m/s)**	Section	<0.001	1	0.872	Large
	Slope	<0.001	1	0.949	Large
	Interaction	<0.001	1	0.654	Large
**Mechanical vertical COM power (W/kg)**	Section	<0.001	1	0.776	Large
	Slope	<0.001	1	0.972	Large
	Interaction	<0.001	1	0.452	Large
**Net metabolic power (W/kg)**	Section	<0.001	0.993	0.600	Large
	Slope	<0.001	1	0.489	Large
	Interaction	<0.001	0.991	0.243	Large
**Net mechanical efficiency**	Section	<0.001	1	0.626	Large
	Slope	<0.001	1	0.969	Large
	Interaction	<0.001	0.994	0.379	Large
**Net metabolic cost of transport (J/kg/m)**	Section	<0.001	1	0.706	Large
	Slope	<0.001	1	0.952	Large
	Interaction	<0.001	0.997	0.406	Large
**Vertical net metabolic cost of transport (J/kg/m)**	Section	<0.001	1	0.648	Large
	Slope	<0.001	1	0.972	Large
	Interaction	<0.001	0.964	0.304	Large

**Table 5:** Differences in values between sections and inclines.

		Sections 1 vs. 2	Sections 1 vs. 3	Sections 2 vs. 3
**Vertical velocity (m/s)**	5°	=0%	↓21.4% *	↓21.4% *
	10°	↓8.33%	↓13% *	↓4.35%
	15°	↓29.6% **	↓35.6% **	↓3.85%
	20°	↓13.3% *	↓36% **	↓20% *
**Velocity (m/s)**	0°	↓51.3% **	↓43.1% **	↑5.75%
	5°	↓1.53%	↓18.6% *	↓16.8% *
	10°	↓8.6%	↓14.3% *	↓5.3%
	15°	↓31% **	↓35% **	↓3%
	20°	↓13.6% *	↓37% **	↓20.5% *
**Mechanical vertical COM power (W/kg)**	5°	↓1.19%	↓19% *	↓17% *
	10°	↓8.86%	↓14.7% *	↓5.33%
	15°	↓30.5% **	↓34.6% **	↓3.15%
	20°	↓15% *	↓37.4% **	↓19.5% *
**Net metabolic power (W/kg)**	0°	↓4.3%	↓12.6%	↓7.95%
	5°	↓6.10%	↓8.75%	↓2.50%
	10°	↓15.3% **	↓15.3% **	=0%
	15°	↓13.3% **	↓12% **	↑1.21%
	20°	↓7.36%	↓4.17%	↑3.07%
**Net mechanical efficiency**	5°	↑4.25%	↓9.41% *	↓14.1% *
	10°	↑6.47%	↓0.72%	↓5.71%
	15°	↓16.2% *	↓19.2% *	↓2.56%
	20°	↓7.73%	↓32.6% **	↓23.1% *
**Net metabolic cost of transport (J/kg/m)**	0°	↑44.9% **	↑35.1% *	↓7.24%
	5°	↓4.37%	↑9.28% *	↑14% *
	10°	↓5.08%	↓0.81%	↑4.24%
	15°	↑14.3% *	↑22.8% *	↑7.5%
	20°	↑7.47%	↑34.5% **	↑25.1% *
**Vertical net metabolic cost of transport (J/kg/m)**	5°	↓4.42%	↑9.35% *	↑14.2% *
	10°	↓4.96%	↓0.84%	↑4.10%
	15°	↑14.6% *	↑23.2% *	↑7.57%
	20°	↑7.45%	↑34.3% **	↑25% *

% of change in mean values with *p*-values of Bonferroni post hoc. Up and down arrows correspond to increases and decreases respectively; equal symbols indicate no change. * *p*-value < 0.05. ** *p*-value < 0.001.

**Table 6:** Multiple linear regression model for mechanical vertical COM power.

R	R^2^	adR^2^	SEE	*p*	Durbin–Watson		B	SE	Beta	*p*	B		VIF
											LL95%	UL95%	
0.975	0.951	0.942	0.07	<0.001	1.911	α	0.133	0.009	1.243	<0.001	0.113	0.152	1.626
						β	−0.030	0.003	−0.797	<0.001	−0.037	−0.023	1.626
						γ	2.376	0.183		<0.001	1.973	2.779	

R: correlation coefficient, R^2^: determination coefficient; adR^2^: adjusted determination coefficient; SEE: standard error of the estimation; *p*: significance level; LL95%: lower limit for 95% confidence interval; UL95%: upper limit for 95% confidence interval, B: multiple linear regression coefficients of each variable; SE: B-standard error; Beta: standardized coefficients; VIF: variance inflation factor; *α*: net metabolic power coefficient; *β:* vertical net metabolic COT coefficient; γ: independent coefficient of the multiple regression.

## Data Availability

The raw data belong to the Universidad San Jorge and can be requested from the corresponding author with the permission of Universidad San Jorge.
